# Sialoadhesin-dependent susceptibility and replication of porcine reproductive and respiratory syndrome viruses in CD163-expressing cells

**DOI:** 10.3389/fvets.2024.1477540

**Published:** 2024-12-24

**Authors:** Hyun-Ji Lee, Su-Hwa You, Hyang-Sim Lee, Yeun-Kyung Shin, Yun Sang Cho, Tae-Sub Park, Seok-Jin Kang

**Affiliations:** ^1^Viral Diseases Research Division, Animal and Plant Quarantine Agency, Gimcheon, Gyeongsangbuk-do, Republic of Korea; ^2^Graduate School of International Agricultural Technology and Institute of GreenBio Science and Technology, Seoul National University, Pyeongchang-gun, Gangwon-do, Republic of Korea

**Keywords:** porcine reproductive and respiratory syndrome virus, Sialoadhesin, CD163, cell line, gene expression

## Abstract

Understanding the molecular interactions between porcine reproductive and respiratory syndrome viruses (PRRSVs) and host cells is crucial for developing effective strategies against PRRSV. CD163, predominantly expressed in porcine macrophages and monocytes, is a key receptor for PRRSV infection. CD169, also known as Sialoadhesin, has emerged as a potential receptor facilitating PRRSV internalization. In this study, we investigated PRRSV susceptibility in relation to CD169 expression in CD163-expressing cells. Susceptibility to PRRSV infection was estimated by immunostaining the N protein using SR30A and quantifying ORF7 using RT-PCR. PRRSV strains adapted to MARC-145 did not infect CD163+/CD169-cells but successfully replicated in CD163+/CD169+ cells. Similarly, porcine alveolar macrophage-isolated PRRSV strains effectively infected and propagated in CD163+/CD169+ cells compared to CD163+/CD169-cells (100% vs. 82.9%). We confirmed that high CD169 expression in CD163-expressing cells increases susceptibility to PRRSVs compared to low or no CD169 expression. In conclusion, CD169 expression level influences viral entry efficiency into CD163-expressing cells, providing valuable insights for isolating wild PRRSVs and producing high-titer PRRS vaccine candidates.

## 1 Introduction

Porcine reproductive and respiratory syndrome virus (PRRSV) is a highly contagious pathogen that significantly affects the swine industry by targeting the reproductive and respiratory systems of pigs. PRRSV belongs to the family *Arteriviridae* and possesses a positive-sense single-stranded RNA genome (approximately 15 kb in length) that encodes 10 open reading frames (1, 2). Infected pigs exhibit symptoms such as fever, respiratory distress, lethargy, and abortion. The virus spreads via direct contact with respiratory secretions and can be fatal, particularly in piglets and pregnant sows, resulting in substantial financial losses to the swine industry.

Porcine alveolar macrophages (PAMs) and monkey kidney cells, such as MARC-145, are commonly used for PRRSV propagation. In the natural host, pigs, the primary targets of PRRSV infection are CD163+ macrophages. Although primary PAMs are widely utilized for *in vitro* pathological research and the isolation of PRRSV, their acquisition and long-term use are challenging. Additionally, heterogeneous cell population during the PAM recovery from individual pigs or across different ages may introduce potential complication. Conversely, MARC-145 cells are favored for vaccine antigen production. However, MARC-145 cells, which are the monkey-derived heterologous cells and the absence of PAM characteristics make them unsuitable for certain PRRSV studies due to the possibility of genetic alterations occurring during the cell adaptation process of PRRSV and the accidental infection of PRRSV, which is a different mechanism with cellular receptor-mediated infection in PAM.

Sialoadhesin (CD169) and CD163 are the two main cellular receptors facilitating PRRSV entry into porcine macrophages (3–5). Although the relationship between CD163 expression and PRRSV infection has been investigated in PAMs (6), and the introduction of the CD163 gene into PRRSV-non-susceptible cell lines has rendered these cells permissive to PRRSV infection (7), the role of CD169 in enhancing susceptibility to PRRSV, particularly in CD163-expressing cell lines, remains underexplored. Previous studies have primarily focused on introducing various cellular receptors, including CD163 and CD169, to augment susceptibility to PRRSV (8).

Several studies have reported that CD163 expression confers susceptibility to PRRSV (4, 5); however, there may be differences depending on the PRRSV strain and infection titer. Although the PRRSV uncoating process involving CD163 is essential for replication in infected cells, it seems that the expression of CD169 is necessary for inducing a higher susceptibility because Sialoadhesin facilitates the intracellular internalization of PRRSV by binding to the viral envelop protein (GP5 and membrane protein) (3, 9). Therefore, we aimed to investigate the impact of CD169 expression on susceptibility to PRRSV infection using CD163-expressing cell lines. Our objective was to develop efficient PRRSV-susceptible pig cell lines, providing valuable insights for isolating wild PRRSVs and producing high-titer PRRS vaccine candidates.

## 2 Materials and methods

### 2.1 Cells

The 3D4/31 PAM cell line (ATCC, CRL-2844) was cultured according to standard procedures. Briefly, 3D4/31 cells were maintained and sub-passaged in DMEM (Invitrogen, Carlsbad, CA, USA) supplemented with 10% fetal bovine serum (FBS, Invitrogen) and 1× antibiotic-antimycotic solution (Invitrogen). The cells were cultured in an incubator at 37°C in an atmosphere of 5% CO_2_ and 60–70% relative humidity. Primary cells were isolated and harvested from pig liver (IACUC permission number 2023–762). As control cells, MARC-145 cells and primary PAMs were cultured in RPMI1640 containing 10% FBS and 1× antibiotic-antimycotic solution. All cells were maintained at 37°C with 5% CO_2_ in a humidified incubator.

### 2.2 Gene transfection

To develop immortalized cells, the piggyBac-based SV40 large T-antigen expression plasmid was transfected into primary cells from the pig liver. For lipofection, SV40 large T antigen and piggyBac transposase expression vector were co-transfected at a ratio of 1:1 (2.5 μg, 2.5 μg) using Lipofectamine® 3000 (Invitrogen) according to the manufacturer’s instructions. Stably growing cells were selected using a puromycin-resistant gene supplemented with 4 μg/mL puromycin for more than 2 months. Pig CD163 (NCBI accession number, AJ311716) and CD169 (NCBI accession number, XM_021077303) genes were synthesized (Bioneer Inc., Daejeon, Korea), and the expression vectors controlled by cytomegalovirus (CMV) immediate-early enhancer/promoter were constructed followed by insertion between the 5′-terminal repeat (5′-TR) and 3′-terminal repeat (3′-TR) piggyBac transposon elements (System Biosciences, Palo Alto, CA). Pig CD163 expression vector and piggyBac transposase expression vector were co-transfected as described above, and stably CD163-expressing sublines were selected using a puromycin-resistant gene supplemented with 10 μg/mL puromycin. Using the selected CD163-expressing sublines, the porcine CD169 expression vector was co-transfected with piggyBac transposes expression vectors and selected using a neomycin-resistant (Neo^R^) gene supplemented with 300 μg/mL G418. Finally, stable porcine cell lines of CD163+/CD169-and CD163+/CD169+ were established.

### 2.3 Viruses

Strains LV, E38, and CBNU0495 as PRRSV-1 were used in this study. LV (GenBank No: M96262) is a prototype of PRRSV-1. The E38 strain (GenBank No: KT033457), which belongs to subtype 1A of PRRSV-1, was isolated from swine on a Korean farm in 2007. CBNU0495 (GenBank No: KY434183) was isolated from the lungs of PRRS-affected pigs, has high pathogenicity and belongs to subtype 1A. The LMY, NA8, and SG strains are PRRSV-2 isolates. LMY (GenBank No: DQ473474) is a vaccine strain that belongs to lineage 1 of PRRSV-2. The NA8 (GenBank No: MZ287322) and SG (GenBank No: PQ777357) strains were isolated from swine on a Korean farm in 2017 and 2022, respectively. Generally, wild type PRRSVs are isolated in PAMs, and a low ratio of PAM-isolated strains can be adapted in MARC-145 cells. Four strains (LV, E38, NA8, and LMY) were adapted in MARC-145, whereas CBNU0495 and SG were propagated only in PAMs.

### 2.4 Clinical samples

Clinical samples were collected from pigs with reproductive failure and piglets with respiratory diseases from different regional provinces in the Republic of Korea in 2022. These samples were kindly provided by the Harim Bio-Research Center (Daejeon, Korea). Tissue samples were homogenized by adding 1× phosphate-buffered saline (PBS) containing 1× antibiotic-antimycotic solution, centrifuged for 5 min at 5,000 rpm, and the supernatant was stored at −80°C until use. Serum samples were used directly in subsequent experiments.

### 2.5 Flow cytometry

For flow cytometry analysis, the cells were resuspended in 1× PBS containing 1% bovine serum albumin (BSA) and strained using a 40-μm cell strainer (Becton, Dickinson and Company, Franklin Lakes, NJ) after incubation with mouse anti-pig CD163 (clone# 2A10/11, Bio-Rad, Hercules, CA) or CD169 (clone# 3B11/11, Bio-Rad) monoclonal antibody. Goat Alexa Fluor 488™-conjugated secondary antibody-positive cells were analyzed and sorted using the FACSAria III cell sorter (Thermo Fisher Scientific). Briefly, pig CD163+/CD169+ cells cultured in a 25 T-flask were harvested by treatment with 0.25% Trypsin–EDTA (Invitrogen), fixed with 4% paraformaldehyde, and blocked with 5% donkey serum in 1× PBS for 1 h prior to incubation with the primary antibody (1:10 for anti-pig CD163 and 1:200 for anti-pig CD169). After incubation with primary antibodies for 1 h, the cells were incubated for 1 h with Alexa Fluor 488™ goat anti-mouse IgG secondary antibody (1:200) (Invitrogen, Waltham, MA) and then washed with 1× PBS. Immunofluorescence was detected under a fluorescence microscope before flow cytometry analysis.

### 2.6 Immunocytochemistry

For immunostaining of PRRSVs, an SR30A monoclonal antibody (Rtilab, Livonia, MI, USA) was used as the primary antibody to detect the N-protein translated from ORF7. Briefly, 3D4/31-CD163, 3D4/31-CD332, pL163, pL332, MARC-145, and PAMs were infected with each strain at a MOI of approximately 0.01 for 2 h, and then washed twice with 1 × PBS. After 72 h, PRRSV-positive cells were determined by immunofluorescence staining with a PRRSV-specific SR30A primary antibody (1:200) for 1 h and Alexa Fluor 488™ goat anti-mouse IgG secondary antibody (1:200) for 1 h. Nuclei were stained with DAPI (10 μg/mL) at room temperature for 5 min. Finally, the cells were analyzed using an EVOS FL Auto2 (Invitrogen). Positive cells were counted and expressed as fluorescent foci units (FFU).

### 2.7 Quantitative real-time reverse transcription (RT)-polymerase chain reaction (PCR)

Total RNA was extracted using a Maxwell RSC Viral Total Nucleic Acid Purification Kit, according to the manufacturer’s instructions (Promega, Madison, WI, USA). The RNA was then used to synthesize cDNA. The cDNA samples were initially subjected to multiplex PCR using genotype-specific ORF7 primers and a one-step real-time RT-PCR kit (GeNet Bio, Daejeon, Korea) to amplify PRRSV-1 and -2 under the following reaction conditions: at 50°C for 20 min (1 cycle), pre-denaturation at 95°C for 10 min (1 cycle), and denaturation at 95°C for 10 s and annealing/extension at 60°C for 30 s (40 cycles). The threshold cycle (Ct) value was calculated as the copy number using the standard curve.

### 2.8 Western blot

Cells were seeded in T75-flasks at a density 1 × 10^6^ cells/mL with 10 mL of medium and then incubated for 72 h at 37°C. The cells were harvested and washed twice with 1× DPBS buffer (CureBio, Seoul, Korea). Each sample was centrifuged for 5 min at 5,000 rpm, and the pellet was stored at −80°C. The cells were lysed with 2× Laemmil’s sample buffer (GenDEPOT, Baker, TX, USA) supplemented with 1× protease inhibitor cocktail solution (GenDEPOT). The 2× Laemmil’s sample buffer was used under both reducing and non-reducing conditions. Samples were loaded onto a 12% polyacrylamide gel and electrotransferred to a polyvinylidene difluoride (PVDF) membrane, which was blocked with skimmed milk (5%) at room temperature for 1 h. Subsequently, the membrane was incubated with primary antibodies (diluted in 3% BSA in TBS-T) at 4°C overnight. The primary antibodies (1:400) used were mouse anti-pig CD163 and mouse anti-pig CD169. The membrane was washed twice with TBS-T for 20 min and then incubated with horseradish peroxidase-labeled secondary antibody (anti-mouse, Santa Cruz, SC-516102) (1:5,000) for 1 h at room temperature. Protein bands were visualized using enhanced chemiluminescence (10) substrate solution (Thermo Fisher Scientific).

### 2.9 Statistical analysis

The results are expressed as the mean ± SD for triplicate experiments (*n* = 3). Statistical significance was determined using a t-test or one-way analysis of variance with Dunnett’s multiple comparison test, and the analyses were performed using GraphPad Prism 7 software (GraphPad Software, Inc., San Diego, CA, USA). A *p*-value <0.05 was considered statistically significant.

## 3 Results

### 3.1 Establishment of immortalized cell lines expressing CD163 only or CD163/CD169 together

**Figure 1:**
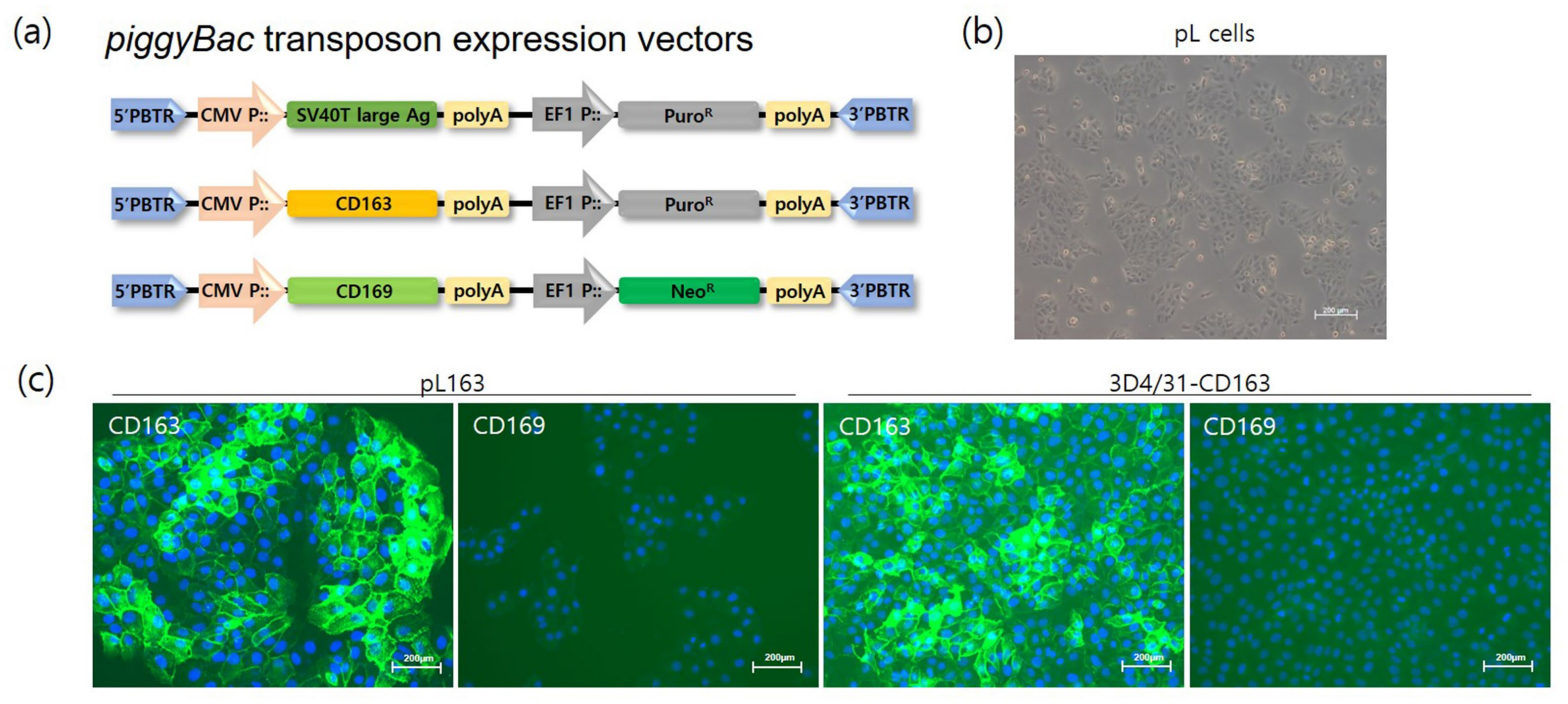
Development of pig immortalized cells expressing CD163. **(A)** Transgenes (SV40T large-antigen, CD163 and CD169 genes) were loaded onto a *piggyBac* transposon expression vector. The CMV and EF1 promoters controlled the expression of the transgenes and the drug resistance genes, respectively. The 5’-PBTR and 3’-PBTR elements indicate the *piggyBac* transposon elements. **(B)** Phenotype of pL cells after the establishment of immortalized cells by introducing SV40T large antigen and puromycin selection (magnification bar = 200 μm). **(C)** The CD163 gene was transfected into pL and 3D4/31 cells, and single cell-derived pL163 and 3D4/31-CD163 cells were propagated under puromycin treatment. Stable expression of CD163 were confirmed by immunostaining of pL163 and 3D4/31-CD163 but not that of CD169 (magnification = 200×).

The establishment of cell lines focuses on developing lines that can be stably and continuously maintained *in vitro*, ensuring consistent experimental conditions and improving the reproducibility of results. This study aimed to develop a susceptible cell line for the reliable acquisition and stable production of PRRSV field isolates. Typically, immortalized cells are generated by introducing the SV40 and hTERT (human Telomerase Reverse Transcriptase) genes. Here, to develop immortalized cells, porcine primary liver cells were co-transfected with a piggyBac-based SV40 large T-antigen and piggyBac transposase expression vector (Figure 1A). After selection with puromycin, continuously growing cells were established and designated as pL cells (Figure 1B). These immortalized pL cells proliferated stably, even after more than 20 passages (data not shown). To establish PRRSV-susceptible cell lines, CD163, one of the key cellular receptors, loaded onto a piggyBac-based expression vector was introduced into pL and 3D4/31 cells (Figure 1A). Although 3D4/31 cells originate from PAMs, they do not express PRRSV cellular receptors, such as CD163 and CD169. CD163-expressing cells (CD163+/CD169-) were selected using high concentrations of puromycin, and CD163 expression was confirmed in pL163 and 3D4/31-CD163 cells (Figure 1C).

**Figure 2:**
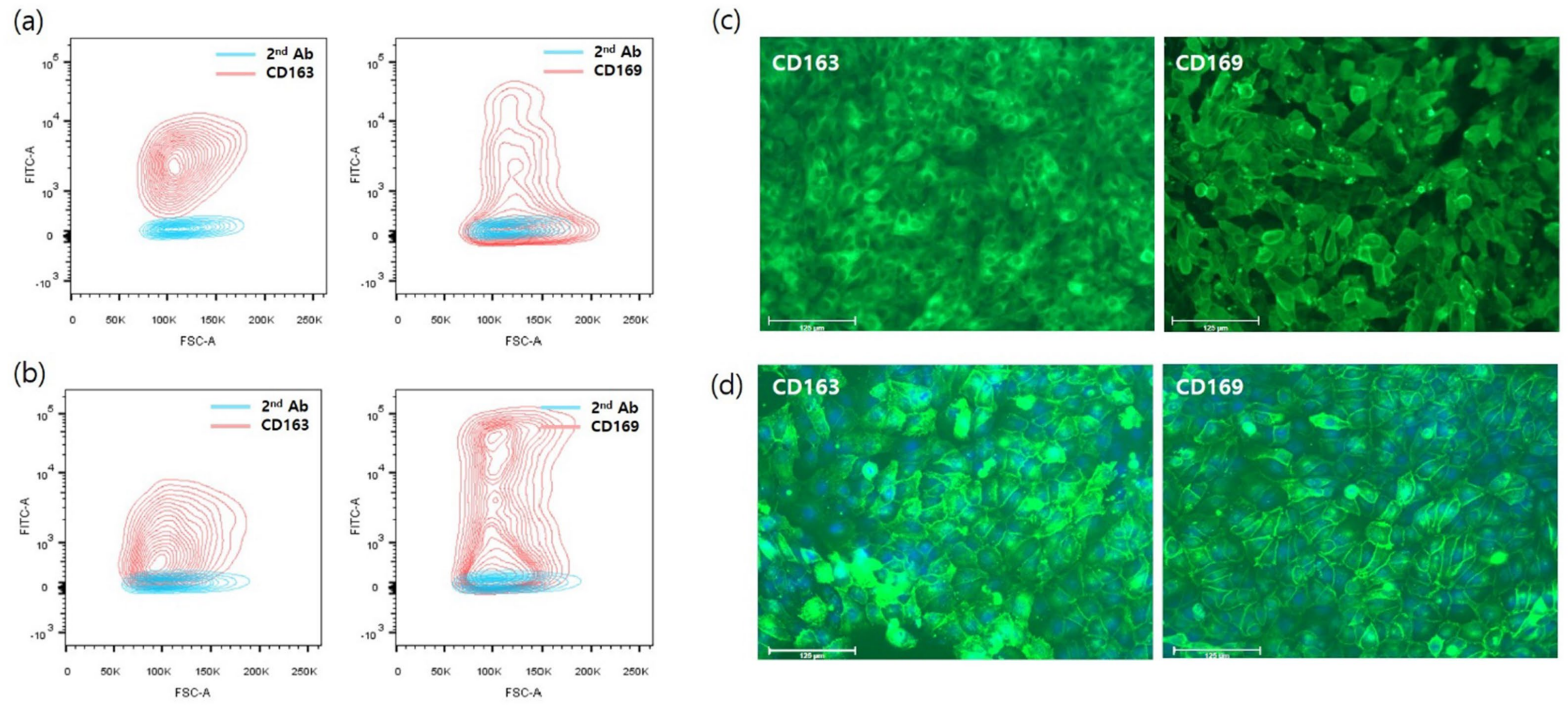
Flow cytometry analysis and immunofluorescent staining with pig CD163 or CD169 antibody. To generate cells that simultaneously express CD169 in CD163-expressing cells, CD169 gene loaded on *piggyBac* transposon expression vector (Figure 1A) was introduced into pL163 and 3D4/31-CD163 cells, which stably express CD163. The CD163 and 169-double positive cells were sorted using a FACSAria III cell sorter in pL163 **(A)** and 3D4/31-CD163 **(B)**. Single cell-derived 3D4/31-CD332 **(C)** and pL332 **(D)** stably expressed CD163 and CD169.

To establish CD163+/CD169+ cells, CD169 was introduced into CD163-expressing 3D4/31-CD163 and pL163 cells using the same procedure. These cells were defined as pL332 and 3D4/31-CD332, respectively. Stable expression of porcine CD163 or CD169 was analyzed using flow cytometry, and cells highly expressing CD163 or CD169 were sorted by FACS (Figures 2A,B). In addition, immunofluorescence staining with anti-porcine CD163 or CD169 antibodies was performed to verify the expression of porcine CD163 or CD169. Pig CD163 and CD169 were stably and highly expressed in both 3D4/31-CD332 and pL332 cells (Figures 2C,D).

### 3.2 Susceptibility of cell lines to PRRSV

**Figure 3:**
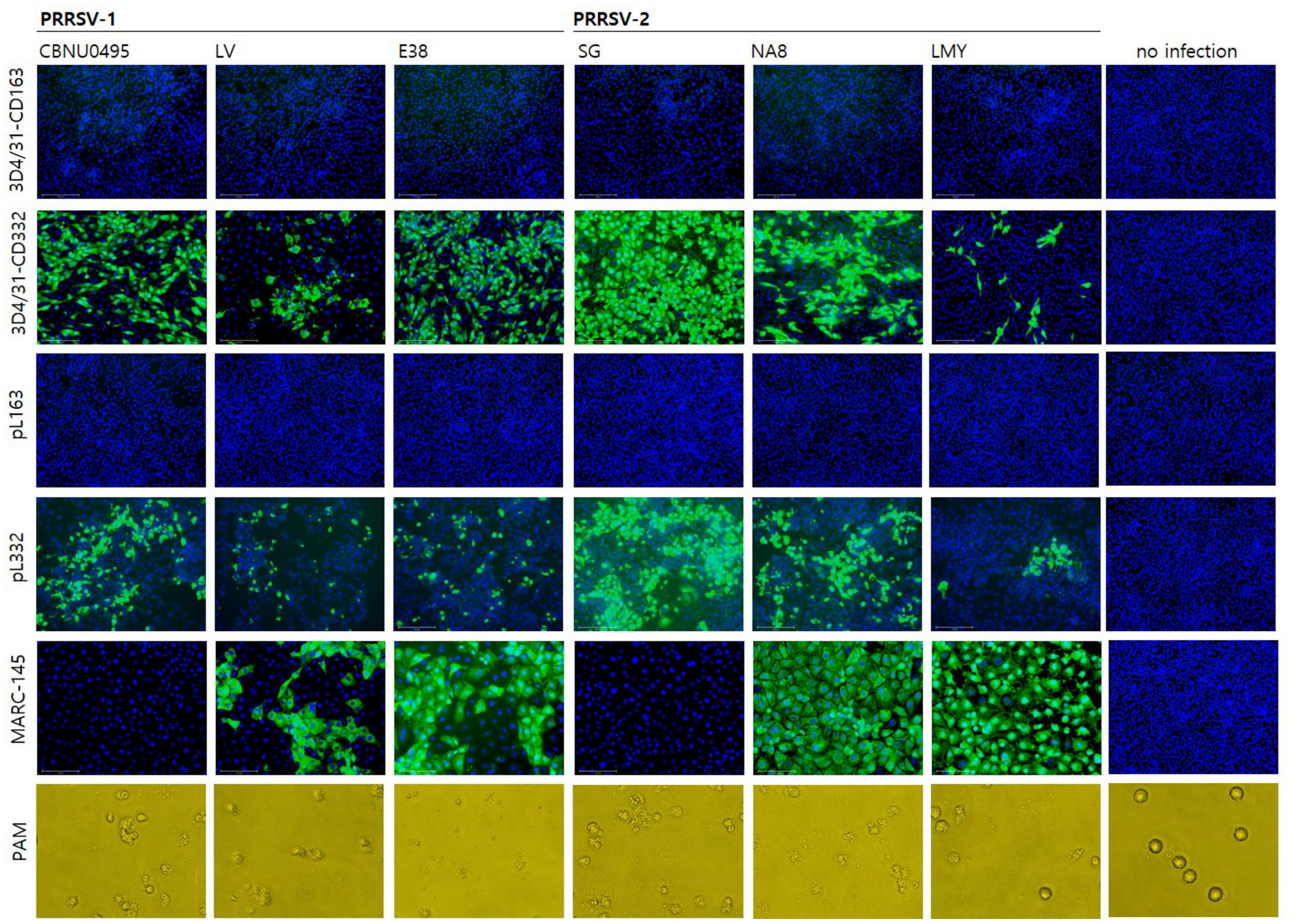
Evaluation of susceptibility to PRRSV in cell lines. The susceptibility of PRRSV-1 (CBNU0495, LV and E38) and PRRSV-2 (SG, NA8 and LMY) was investigated in CD163+/CD169- (3D4/31-CD163 and pL163) and CD163+/CD169+ cell lines (3D4/31-CD332 and pL332), as well as MARC-145 and PAM cells as controls. At 3 days post-infection, PRRSV susceptibility was confirmed by immunostaining for the N protein (SR30A) in 3D4/31-CD163, 3D4/31-CD332, pL163, pL332 and MARC-145 cells, and by cytopathic effect (CPE) in PAMs.

**Figure 4:**
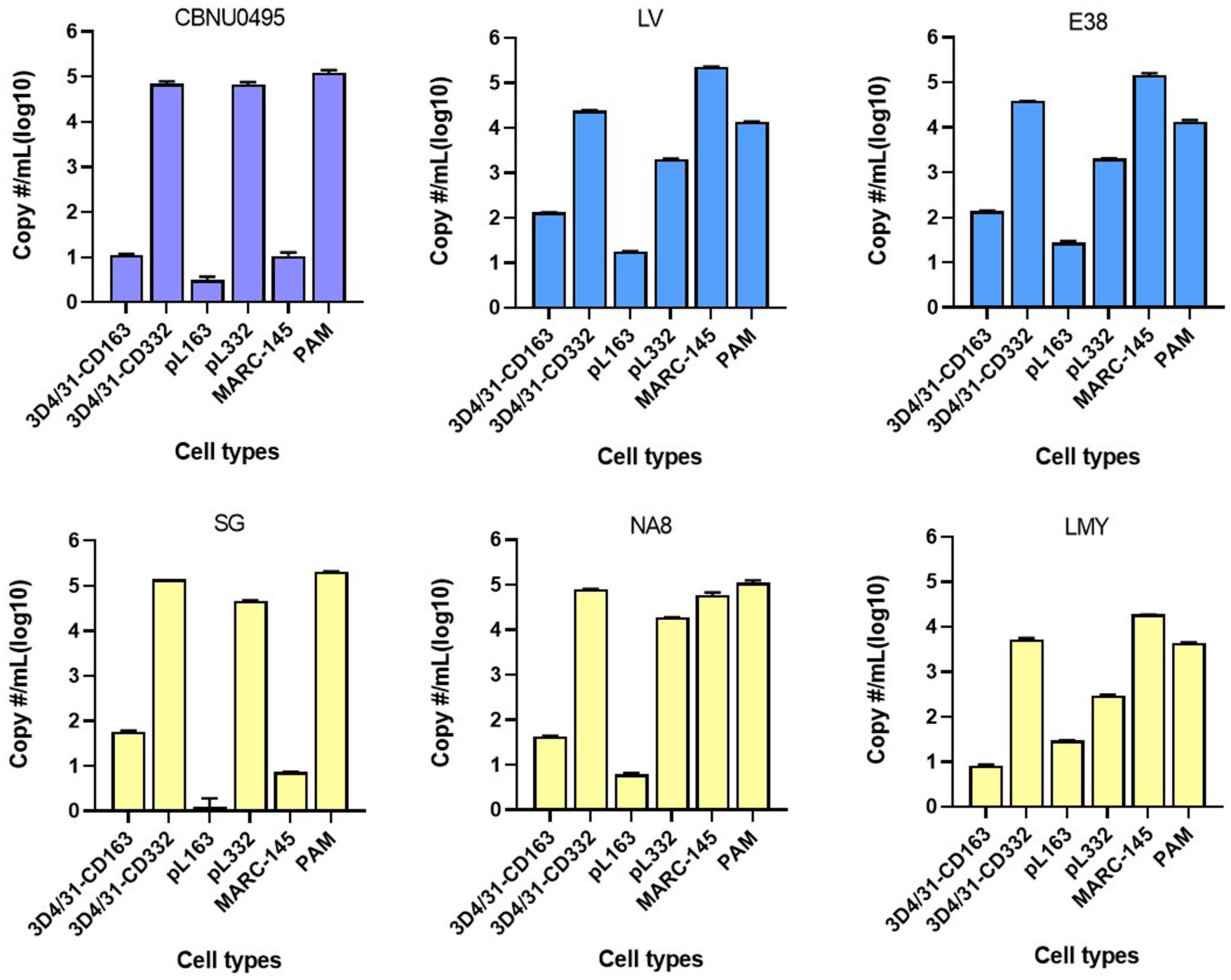
Estimation of RRSV infectivity in cell lines. To estimate viral infectivity of six cells (3D4/31-CD163, 3D4/31-CD332, pL163, pL332, MARC-145 and PAMs), six PRRSV strains (CBNU0495, LV and E38 of PRRSV-1, and SG, NA8 and LMY of PRRSV-2) were used to infect cells, and genomic copy numbers of PRRSVs were measured by qPCR at 4 days post-infection. All strains replicated in CD163+/CD169+ cell lines (3D4/31-CD332 and pL332) and PAMs, but not in CD163+/CD169-cells (3D4/31-CD163 and pL163). MARC-145-adapted strains (LV, E38, NA8, and LMY), except PAM-isolated PRRSVs (CBNU0495 and SG), propagated in MARC-145.

To investigate the susceptibility of cells to PRRSV, six PRRSV strains were used to infect six cell lines. CBNU0495 and SG were propagated in PAMs but not MARC-145 cells, whereas LV, E38, NA8, and LMY were propagated in MARC-145 cells. As shown in Figure 3, all PRRSV strains induced a cytopathic effect in PAMs; however, CBNU0495 and SG did not infect MARC-145, unlike the other four strains (LV, E38, NA8, and LMY). No PRRSV infection was observed in CD163-expressing cell lines (3D4/31-CD163 and pL163). In contrast, all strains infected CD163+/CD169+ cell lines (3D4/31-CD332 and pL332). These results were confirmed by quantifying the genomic copy numbers of the six PRRSV strains in the six different cell types (Figure 4). All six strains propagated in PAMs, and four strains, except CBNU0495 and SG, replicated in MARC-145. In cells transformed with PRRSV cellular receptors (CD163 and CD169), all six strains highly propagated in CD163+/CD169+ cells (3D4/31-CD332 and pL332), but not in CD163+/CD169-cells (3D4/31-CD163 and pL163). These data were consistent with the immunofluorescence assay results (Figure 3). Thus, CD169 is a critical receptor for PRRSVs in CD163-expressing cells.

### 3.3 Effects of CD169 expression on PRRSV replication and susceptibility

**Figure 5:**
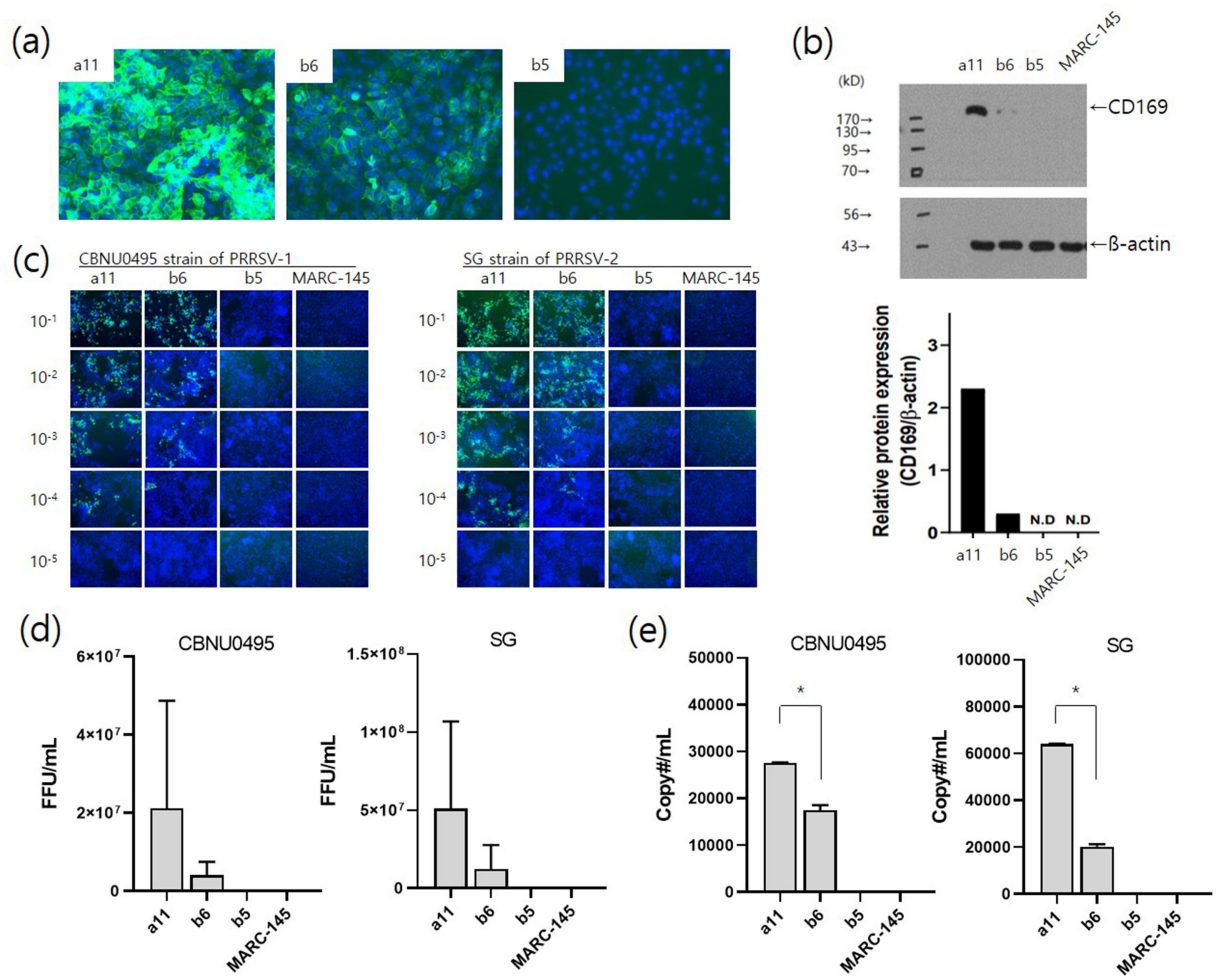
Effects of CD169 expression on PRRSV infection. Three clones (a11, b6, and b5) of pL332 were selected based on CD169 expression levels: a11^high^, b6^low^ and b5^no^. The expression intensity of CD169 was confirmed by immunofluorescent staining **(A)** and western blotting **(B)** with pig anti-CD169. The pL332-a11 clone showed strong fluorescence intensity and protein expression. The pL332-b6 and -b5 clones showed moderate and negative levels, respectively. Susceptibility to PRRSV was confirmed by immunostaining for the N protein (SR30A mAb) in pL332-a11^high^, −b6^low^, −b5^no^, and MARC-145 after infection with serial dilutions (10^−1^ to 10^−5^) of PRRSV-1 (CBNU0495) and PRRSV-2 (SG) **(C)** and viral titers (FFU/mL) of CBNU0495 and SG were estimated by counting SR30A-positive colonies at 3 days post-infection **(D)**. Genomic copy numbers of PRRSVs were measured by qPCR at 4 days post-infection **(E)**. MARC-145 cells were used as a negative control. * indicates a significant difference (*p* < 0.05).

To investigate the effects of CD169 expression on PRRSV infection, single cell-derived clones were selected from pL332 parent cells based on CD169 expression levels. The selected clones were pL332-a11 (high CD169 expression), pL332-b6 (low CD169 expression), and pL332-b5 (no CD169 expression). CD169 expression levels were confirmed by fluorescence intensity (Figure 5A), and protein expression was verified using western blot (Figure 5B). In terms of viral susceptibility, the pL332-a11^high^ clone was more susceptible to CBNU0495 and SG strains than the pL332-b6^low^ clone. PRRSV strains did not infect the pL332-b5^no^ clone or MARC-145 (Figure 5C). These results were further confirmed by measuring fluorescent foci units (FFU/mL) (Figure 5D) and quantifying genomic copy numbers (copy#/mL) (Figure 5E).

### 3.4 Effective isolation of PRRSVs from clinical samples

**Figure 6:**
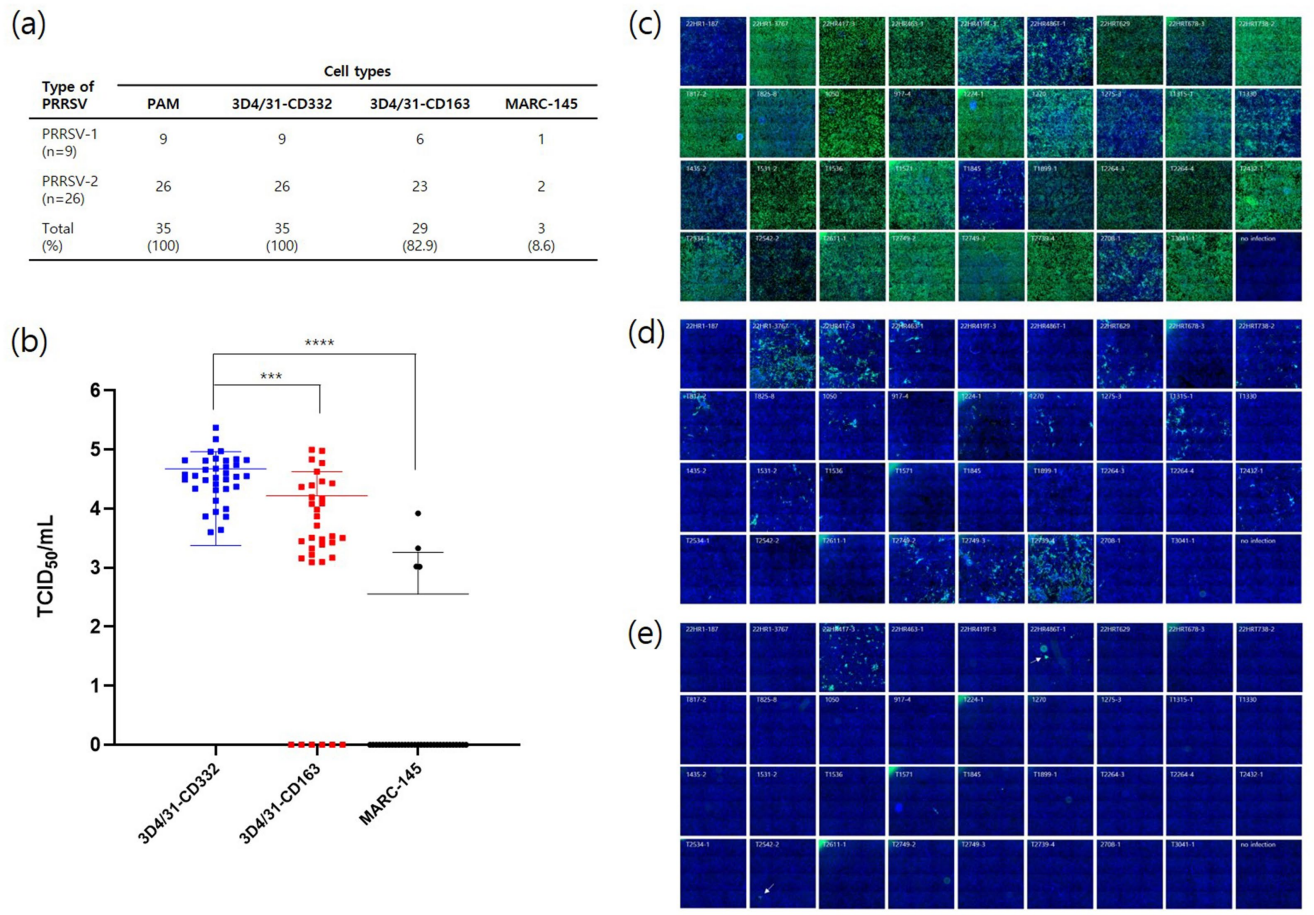
Isolation of field PRRSVs from different cell lines. Using 35 PRRSV-positive clinical samples, PRRSVs were isolated from PAMs, 3D4/31-CD332, 3D4/31-CD163, and MARC-145 **(A)**. Viral titers were measured from the supernatants of each cell line by qPCR **(B)**. PRRSVs were isolated as 35 (100%), 35 (100%), 29 (82.9%) and 3 (8.6%) in PAMs, 3D4/31-CD332, 3D4/31-CD163, and MARC-145, respectively. PRRSVs were immunostained with SR30A mAb in 3D4/31-CD332 **(C)**, 3D4/31-CD163 **(D)**, and MARC-145 **(E)** at 3 days post infection. The fluorescent intensity was the highest in 3D4/31-CD332 **(C)** and was consistent with the result of qPCR **(B)** ***, **** indicates a significant difference (*p* < 0.001 and *p* < 0.0001, respectively).

To isolate PRRSV-1 (*n* = 9) and PRRSV-2 (*n* = 26), 35 clinical samples positive for PRRSVs were used to infect three cell lines: 3D4/31-CD332, 3D4/31-CD163, and MARC-145. Wild-type PRRSVs from all clinical samples were isolated in 3D4/31-CD332 cells (100%) (Figure 6A). The isolation efficiencies for 3D4/31-CD163 and MARC-145 were 82.9% (29/35) and 8.6% (3/35), respectively (Figure 6A). Moreover, the viral titers in 3D4/31-CD332 were significantly higher (*p* ≤ 0.001) than those in 3D4/31-CD163 and MARC-145 cells (Figure 6B). These differences in viral titers were confirmed by immunofluorescent intensity for the N protein of PRRSV in 3D4/31-CD332 (Figure 6C), 3D4/31-CD163 (Figure 6D), and MARC-145 cells (Figure 6E).

## 4 Discussion

This study aimed to develop efficient porcine cell lines with susceptibility to and replication ability for PRRSV. Currently, PAMs are used to isolate wild-type PRRSV, while MARC-145 cells are employed to produce vaccine antigens. However, both types of cells have significant limitations. PAMs must be isolated from pig lungs and cannot proliferate *in vitro*, limiting their use to a single instance. MARC-145 cells, derived from monkey kidney cells, present challenges, such as being a heterologous cell line, being infected by PRRSV through mechanisms different from those in host cells, and having low efficiency in isolating field strains. To address these issues, researchers have introduced various cell receptor genes, including CD163, CD169 (or SIGLEC-1), and CD151, which are recognized as PRRSV receptors (8). To date, nine PRRSV cell receptors have been reported: CD163, a cysteine-rich scavenger receptor, heparin sulfate, Sialoadhesin (CD169 or SIGLIC-1), cluster of differentiation 151 (CD151); vimentin, dendritic cell-specific intercellular adhesion molecule-3-grabbing non-integrin (DC-SIGN), non-muscle myosin heavy chain 9 (MYH9), and heat shock protein member 8 (11–15).

The scavenger receptor—CD163—plays a critical role in PRRSV infection by binding to the PRRSV surface GP2a/3/4 complex and promoting viral internalization and uncoating in macrophages (7). CD163-knockout pigs are not susceptible to PRRSV (5, 16). Similarly, CD163-deficient cells do not produce PRRSV progeny, and the introduction of CD163 into these cells restores their susceptibility (6). However, in this study, infection and proliferation of two PAM-isolated viruses (SG and CBNU) and four MARC-145-isolated viruses (LV, E38, NA8, and LMY) were not observed in the CD163-only expressing cell line (Figures 3, 4). In addition, the efficiency of PRRSV infection in CD163-expressing cells was significantly lower (Figure 6). These results differ from previous studies (5–7, 16) reporting that non-permissive cells became susceptible to PRRSVs after only CD163 introduction. This discrepancy may be due to the relatively low levels of CD163 expression in the generated cells. Although we confirmed stable CD163 overexpression in single cells, the CD163 fluorescence intensities of the cells were very low. It remains unclear whether this was due to the low affinity of porcine anti-CD163 or the relatively weak expression of CD163. The limited availability of porcine CD163 antibodies made additional validation difficult. However, it can be inferred that PRRSV is produced when CD169 is introduced into CD163 cell lines with stable CD163 expression. Additionally, it is necessary to evaluate the susceptibility and replication of PRRSVs based on the expression levels of CD163.

CD169 is a membrane glycoprotein belonging to the Siglec family of sialic acid-binding immunoglobulin-like lectins that stimulate the internalization process (17). It is found on the surface of immune cells, particularly macrophages and dendritic cells (18). CD169 binds to sialic acid residues on the cell surface, thereby affecting immune cell function and interactions with viruses or other cells. This receptor can detect PRRSV particles and potentially facilitate their absorption by interacting with sialic acid residues on the cell surface (19, 20). It plays a role in the internalization of PRRSV into cells by binding to PRRSV M/GP5 (19). In this study, we confirmed that PRRSV replication was enhanced by the introduction of CD169 into CD163-expressing cells (Figure 4). As shown in Figure 5, there was a difference in susceptibility depending on the expression level of CD169, suggesting that increased internalization into cells enhanced replication efficiency (Figure 5). Therefore, not only can the production capacity of PRRSV be improved through the simultaneous expression of CD163 and CD169, but the efficiency of field virus isolation can also be enhanced.

Currently, most PRRS vaccine antigens are produced using MARC-145. As mentioned above, MARC-145 is a monkey kidney-derived cell line that is infected with PRRSV through a mechanism different from that of PAMs (21). Furthermore, PRRSV isolated from PAMs showed low rates of adaptation to MARC-145. In this study, only three (8.6%) of the 35 strains isolated from PAMs were able to infect MARC-145. Additionally, MARC-145-adapted strains do not replicate substantially in PAMs (22). Amino acid substitutions in GP2a (V88F, M94I, and F95L) have been identified in MARC-145-adapted PRRSVs (23). It is speculated that these genetic changes contribute to the reduced PRRSV replication rate in PAMs. Previous studies have shown that MARC-145-adapted PRRSV strains exhibit lower replication kinetics in PAMs than PAM-adapted PRRSV strains (data not shown). Furthermore, their antigenicity is reduced (24), suggesting that the passage number of vaccine antigens should be restricted. Further research is required to investigate the genetic and antigenic alterations in the cell lines developed in this study. Nevertheless, the CD163+/CD169+ cell lines could be used to isolate six strains (Figure 4) and field strains (Figure 6) with efficiencies comparable to those of PAMs.

The current PRRS vaccines are primarily attenuated modified-live vaccines (MLV). While MLV faces challenges regarding safety, such as the potential for reversion to virulence (25, 26) and recombination between vaccine and wild type strains (10, 27), there are additional issues during the development of vaccine candidates. As mentioned above, MARC-145 has low infection efficiency, making it difficult for used to develop vaccine candidates. Inactivated vaccines are safe, but they have the drawback of low efficacy. To improve efficacy, there is a need to induce high antigen levels and obtain effective adjuvants. Producing high levels of antigens requires concentrating the vaccine antigens produced in MARC-145, which increases production costs. Therefore, the development of MLV and inactivated vaccines requires cell lines with high isolation efficiency and productivity. In 2022, the NADC34-like field virus was first detected in South Korea (28). Moreover, the efficacy of current commercial vaccines is low (data not shown). Therefore, there is a need for genotype-specific vaccine development. The cell lines developed herein allow for easy isolation of field strains and enable production with high antigen titers. This can facilitate a rapid response to newly introduced or mutated strains. Therefore, these cells can be utilized as vaccine-producing cell lines. In further studies, it is necessary to evaluate the genetic and antigenic alteration of PRRSVs during production in CD163+/CD169+ cells and to compare with those in PAMs and MARC-145.

In conclusion, the expression of CD169 in CD163-expressing cells enhanced susceptibility to and replication of PRRSVs. Additionally, the efficiency of isolating and propagating field strains in CD163+/CD169+ cells was comparable to that in PAMs. Therefore, CD163/CD169 dual-expressing cells can be utilized in basic PRRSV research and in the production of vaccine antigens in the future.

## Data Availability

The original contributions presented in the study are included in the article/supplementary material, further inquiries can be directed to the corresponding authors.
